# A study of crystalluria: effectiveness of including hygienic-dietary recommendations in laboratory reports

**DOI:** 10.1515/almed-2020-0124

**Published:** 2021-02-10

**Authors:** Paula Sienes Bailo, María Santamaría González, Silvia Izquierdo Álvarez, Raquel Lahoz Alonso, Patricia Serrano Frago, José Luis Bancalero Flores

**Affiliations:** Service of Clinical Biochemistry, Miguel Servet University Hospital, Zaragoza, Spain; Service of Urology, Miguel Servet University Hospital, Zaragoza, Spain

**Keywords:** hygienic-dietary recommendations, nephrolithiasis, renal colic, stones

## Abstract

**Objectives:**

To assess the effectiveness of incorporating hygienic-dietary recommendations in laboratory reports in reducing the incidence of renal colic (RC). A study was performed to compare the incidence of RC in two groups of patients who had suffered at least a crystalluria event associated with the risk of urolithiasis. Recommendations were only incorporated in the laboratory reports of one group.

**Methods:**

A retrospective observational study. The study sample was composed of patients who had at least an episode of crystalluria associated with a higher risk of urolithiasis. The laboratory reports of patients in Group A (n=1,115), treated in 2017, did not include any hygienic-dietary recommendations, whereas patients in Group B (n=1,692), treated in 2018, received hygienic-dietary recommendations through their laboratory reports. χ^2^ and Mann-Whitney U test were used to assess differences based on sex, age, and type of urinary crystals.

**Results:**

The incidence of RC was 2.02 times higher in group A (2.24%) than in group B (1.12%). No significant differences were observed in the incidence of RC based on the type of urinary crystal. The incidence of RC was substantially higher in patients who suffered at least an event of crystalluria associated with a higher risk for urolithiasis as compared to the general population during the same period (0.46%, consistently with the incidence rates reported in the literature).

**Conclusions:**

The incorporation of messages alerting on the risk of urolithiasis and the inclusion of hygienic-dietary recommendations in laboratory reports may be useful for reducing the incidence of RC.

## Introduction

Renal colic (RC) is the most frequent clinical manifestation of urolithiasis. In Spain, it is estimated that 10–20% of men and 3–5% of women will suffer at least an episode of RC at some point in their life, and 30–40% will have recurrent stones in a timeframe of five years. RC is more prevalent in men within the age range of 30–60 years [1]. The frequency of RC increases in the warm season and in the early hours of the morning, is linked to geographical variations, and is dependent on diet and water intake [2]. Based on its composition, oxalocalcium lithiasis is the most common type, followed by uric, mixed (oxalate and calcium phosphate), struvite, and cystine lithiasis [3].

Crystalluria is more frequent in stone formers that in healthy subjects, although crystals can occasionally be found in the urine of healthy subjects. Analysis of urinary sediment is a valuable tool for the detection and monitoring of inherited and acquired diseases associated with the formation of urinary stones, since crystalluria substantially increases the likelihood of their formation [4], [5], [6]. In this sense, the American Urological Association (AUA) guidelines for the management of kidney stones recommend that urinalysis include both the microscopic evaluation of urinary sediment and qualitative analysis to evaluate urinary pH, infection markers and identify pathognomonic crystals of each type of stone [7].

Some types of urinary crystals are associated with an increased risk of renal stone disease based on their chemical and crystal composition, crystalline facies*,* abundance of crystalluria, size of crystals, rate of aggregation and crystal twinning and frequency of crystalluria. Calcium oxalate dihydrate (COD, weddellite) crystals are generally octahedral or bipyramidal, and adopt a dodecahedral conformation [8] when calcium levels in urine increase. Increased levels of calcium in urine raise the risk of stone formation, as calcium is a promotor of crystalization. COD crystals measuring 7–8 μm can be found in healthy subjects, whereas the presence of COD crystals >35 μm indicates active stone formation and a higher risk of recurrence. Likewise, urolithiasis is associated with the presence of uric acid crystals >100 μm. Finally, the presence of calcium oxalate monohydrate (COM, wewellite) and dihydrate in the same urinary sediment, related to concurrent hyperoxaluria and hypercalciuria, increases the risk of lithiasis [9].

In recent years, some authors have drawn attention on the relationship between urolithiasis and diet, more specifically, with low water intake and diets rich in animal proteins, sugar, oxalate and sodium [10], [ 11]. Therefore, including hygienic-dietary recommendations in laboratory reports aimed at patients at a high risk of developing kidney stones may help reduce the incidence of the disease.

The goal of this study was to assess the effectiveness of incorporating hygienic-dietary recommendations in laboratory reports. To such purpose, the incidence of RC was compared in two groups of patients seen in the HUMS during the 2017–2018 period who had suffered at least a crystalluria event associated with a higher risk of stone formation (UA>100 μm, COD>35 μm). Recommendations were only given to one group.

## Materials and methods

### Subjects

A retrospective observational study. Data was extracted from the Hospital Laboratory Information System (Modulab, Werfen, Spain) and the electronic medical records of patients, revised by the physicians of the Section of Renal Function of the HUMS, Zaragoza, Spain, in 2019.

**Figure 1:**
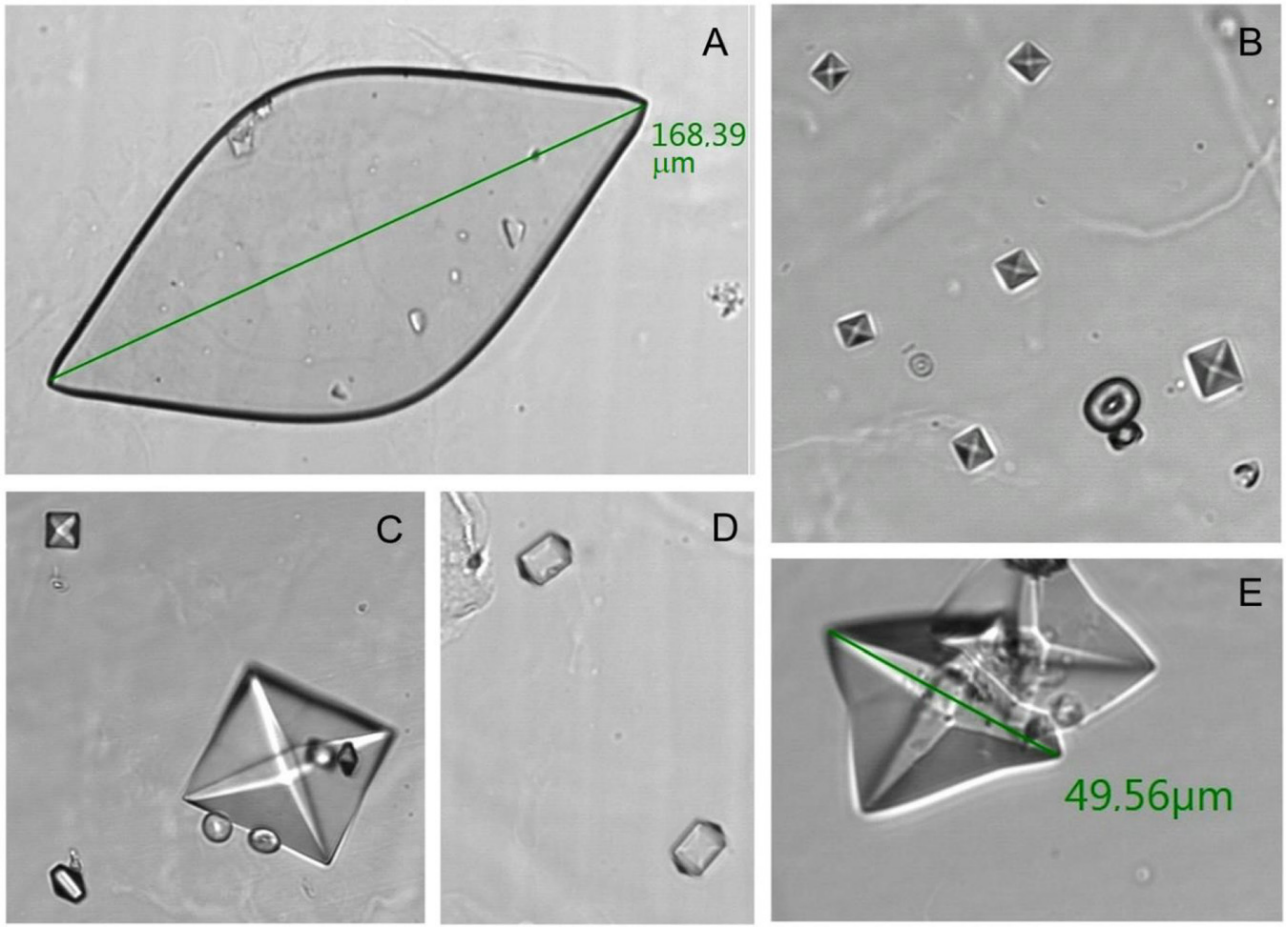
(A–E) SEDIMAX images (A. Menarini Diagnostics, Barcelona, Spain). (A) Uric acid (UA) crystals >100 μm. (B) Calcium oxalate monohydrate (COM) and dihydrate (COD) crystals in the same urinary sediment. (C) COD crystals >35 μm and dodecahedral COD crystals in the same urinary sediment. (D) Dodecahedral COD crystals. (E) COD crystals >35 μm.

Inclusion criteria: patients seen in the HUMS between 2017 (Group A) and 2018 (Group B) with a high risk of urolithiasis. Patients were considered to be at a high risk of utolithiasis if they had a urinary sediment test in 2017 or 2018 and meet at least one of the following criteria (Figure 1) [9]:

- Presence of UA crystals >100 μm (Figure 1A)
- Presence of COD crystals >35 μm (Figure 1C and E)
- Presence of dodecahedral COD crystals (Figure 1 C and D)
- Presence in the same urinary sediment of COD and COM crystals (Figure 1B).

Subjects were divided into two groups according to the information included in their laboratory reports, namely, the reports of one group included a comment that warned of the presence of lithogenic risk, and some hygienic-dietary recommendations for the prevention of RC were proposed to the requesting physician.

– Group A. Patients at a high risk or urolithiasis who were not alerted and whose laboratory reports did not include any hygienic-dietary recommendations. This group included Zaragoza Sector II patients who had at least an episode of crystalluria associated with a higher risk of urolithiasis in 2017.
– Group B. Patients at a high risk or urolithiasis who were alerted on the detected risk of urolithiasis and whose laboratory reports did not include any hygienic-dietary recommendations. This group was composed of patients from Zaragoza Sector II who had at least an episode of crystalluria in 2018 associated with a higher risk of urolithiasis.

Exclusion criteria: patients from other Health Sectors who did not have a urinary sediment analysis in 2017 or 2018 and who did not meet at least one of the criteria associated with a higher risk of urolithiasis described above.

### Methods

First, patients collected samples of first-morning urine in VACUETTE^®^ tubes of 10 mL (Greiner bio-one, Madrid, Spain) and delivered them to the HUMS. Then, the physicians of the HUMS Section of Renal Function performed a urinary sediment analysis using tandem AutionMax AX-4030 and SediMax analyzers (A. Menarini Diagnostics, Barcelona, Spain). The first analyzer performs a first screening using urine reactive strips. If some parameter was found to be altered, SediMax aspirated 2 mL of the sample and transferred 0.2 mL to a cuvette where it was centrifugated at 2,000 rpm for 10 min to perform the urinary sediment analysis. Then, 15 pictures were made at 400× in different fields by bright-field microscopy. Finally, the physicians of the Section of Renal Function evaluated the images obtained and identified, measured and quantified urine components.

The patients who met any of the four criteria associated with a higher risk of urolithiasis were included either in group A or B according to the year they had the test. The patients who had the test in 2017 were included in group A, an alerting comment was not included in their laboratory report and were followed up during 2018 to record the number of RC events. The patients who had the test in 2018 were allocated to group B, and an alert on the risk of urolithiasis addressed to the requesting physician was included in the laboratory report, along with some hygienic-dietary recommendations for the patient (Table 1). These patients were monitored during 2019 to record the number of RC events. The time of follow-up was the same for the two groups (one year).

**Table 1:** Alerts: risk of urolithiasis (A) and hygienic-dietary recommendations (B) included in laboratory reports.

**A**	**Alerts about the risk of urolithiasis**
	(1) Risk of urolithiasis. Presence of some uric acid crystals >100 µm. Assess lithiasis and/or metabolic alteration. Consider implementing hygienic-dietary recommendations.
	(2) Risk of urolithiasis. Presence of some calcium oxalate crystals >100 µm. Active lithogenesis. Consider implementing hygienic-dietary recommendations.
	(3) Risk of urolithiasis. Presence of dodecahedral calcium oxalate crystals. Consider implementing hygienic-dietary recommendations.
	(4) Risk of urolithiasis. Presence of calcium oxalate monohydrate and dihydrate. Consider implementing hygienic-dietary recommendations.
**B**	**Hygienic-dietary recommendations**
	– Increase the intake of liquids to 2–3 L of water daily.
	– Increase the intake of foods rich in phytate and citrate.
	– Maintain the consumption of calcium-rich foods
	– Reduce the intake of oxalate, salt, animal proteins and soft drinks.
	– Avoid calcium and vitamin C and D supplementation.
	– Avoid sedentarism.
(Ask your doctor before suspending any prescribed medication).	

The incidence of RC in the general population was estimated from the total number of RC events recorded in the HUMS Emergency Department in 2017 and 2018 in the Zaragoza II Health District population seen these years (data provided by the HUMS Department of Information Systems). In addition, the incidence of RC was calculated for group A and B based on the number of RC events recorded for each patient and treated in the Service of Renal Function.

This study complies with all national regulations, institutional policies and the ethical principles of the Declaration of Helsinki and was approved by the Ethics Committe of the Autonomous Community of Aragon (CEICA).

### Statistical analysis

For each qualitative variable, the distribution of frequencies was calculated by categories. Differences according to age, which was the only quantitative variable, were explored by the Shapiro-Wilk test (goodness of fit test of normality). Central tendency (median) and dispersion (interquartile range, IQR) were also calculated. χ^2^ and Mann Whitney U test were used to assess differences based on sex, age, incidence of RC, and type of urinary crystals. All statistical analyses were performed using the Jamovi 1.1.9.0 software package. Statistical significance was set at p<0.05 (two tails).

Statistical analysis was complemented with a regression model with the presence or absence of RC as the dependent variable and the parameters with statistically significant differences as independent variables.

## Results

An analysis of urinary sediments was included in 84,342 of the 192,024 urine analyses carried out in 2017. In total, 1,115 patients were included in group A, as they met at least one of the four criteria associated with a higher risk of urolithiasis. Group B was formed using the same inclusion and exclusion criteria, with a final sample of 1,692 patients. Urinary sediments were analyzed in 89,208 of the 196,195 urine analyses carried out in 2018. As many as 25/1,115 (2.24%) patients of Group A and 19/1,692 (1.12%) patients of group B had an episode of RC. Demographic data and types or urine crystals are shown in Table 2.

**Table 2:** Demographic data and types of urinary crystals found.

	Group A 1,115	Group B 1,692	p-Value
**Sex**			0.311
Men	427 (38.3)	616 (36.4)	
Women	688 (61.7)	1,076 (63.6)	
Age at occurrence, years	50.0 ± 25.0	51.0 ± 27.0	0.967
**Range of age, years**			0.467
<13	15 (1.4)	29 (1.7)	
14–29	103 (9.2)	190 (11.2)	
30–45	334 (30.0)	489 (28.9)	
46–64	443 (39.7)	627 (37.1)	
>65	220 (19.7)	357 (21.1)	
**Urinary crystals**			0.017^a^
UA>100 μm	96 (8.6)	126 (7.5)	
COD>35 μm	103 (9.3)	129 (7.6)	
Dodecahedral COD	472 (42.3)	664 (39.2)	
COD + COM	444 (39.8)	773 (45.7)	

Group A: patients whose laboratory reports did not include any alerts or hygienic-dietary recommendations. Group B: patients whose laboratory reports included alerts or hygienic-dietary recommendations. Age is expressed as median ± IQR and sex, age range and types of urinary crystals are expressed as n (%) in terms of absolute and relative frequencies. p-values obtained in comparisons test of medians and proportions are shown in the last column, with significant differences marked with (^a^).COD, calcium oxalate dihydrate; COM, calcium oxalate monohydrate; UA, uric acid.

The two groups were prevailingly composed of women (61.7% *v* 63.6%) without statistically significant differences (χ^2^=1.03; p=0.311). The median age of occurrence of crystalluria was 50 years (IQR 25.0) in Group A patients and 51 years (IQR 27.0) in Group B (p=0.967). Age was not normally distributed in any of the groups (p=0.003 *v* p=<0.001).

Statistically significant differences were observed in distribution by type of urinary crystal associated with a higher risk of urolithiasis (χ^2^=10.2; p=0.017). The presence of UA crystals >100 μm was the least frequent event in the two groups. However, the most frequent findings were the presence of dodecahedral COD crystals in Group A vs. the concurrent presence of COD and COM crystals in Group B.

Table 3A shows the incidence and mean age of occurrence of RC for the two groups. As expected, statistically significant differences were observed in the incidence of RC between group A and B (χ^2^=5.46; p=0.019).

**Table 3:** Incidence and median age at RC occurrence in groups A and B (A) and in the general population (B).

	Group/year of study	RC	Subject/population	Incidence of RC, %	Median age of occurrence, years (IQR)
**(A)**	A	25	1,115	2.24	48.0 (19.0)
	B	19	1,692	1.12	51.0 (20.0)
**(B)**	2017	1,796	390,787	0.46	48.0 (22.0)
	2018	1,776	394,165	0.45	48.0 (23.0)

RC, renal colic; IQR, interquartile range.

The HUMS serves a population of 392,476 from Health District Zaragoza II annually, with a general rate of incidence of RC of 0.46%. Data by year, total number of RC events treated in the HUMS, mean age of occurrence, and incidence of RC (expressed as percentages) are shown in Table 3B.

A binomial regression model was built using the endpoint (presence or absence of RC) as the dependent variable and the inclusion of hygienic-dietary recommendations as factor. The odds ratio was 2.02 (95% CI 1.11–3.68). RC events were 2.02 times more frequent in group A as compared to group B, which received dietary recommendations. There was a statistically significant relationship between the factor (incorporation of hygienic-dietary recommendations for patients at a higher risk of stone formation) and the effect studied (incidence of RC).

## Discussion

In our study, the annual incidence of urolithiasis (expressed as RC) was 0.46% in the general population of the Zaragoza II Sector during the 2018–2019 period, which is consistent with the incidence rates reported in the literature [3], [12], [13].

The incidence of urolithiasis in Spain in 2007 was 0.73%, with a prevalence of 5.06% [3]. In contrast, the prevalence of urolithiasis was 13.9% in a study conducted three years later to assess the prevalence of chronic kidney failure in our country [12], which is in agreement with the prevalence of 14.6% reported in the PreLiRenE study [13]. A higher prevalence (16.3%) was documented in the PreLiRenA study assessing the prevalence of renal lithiasis in the Andalusian population with ages between 40 and 65 years [14]. In the PreLiRenE study, the rate of prevalence ranged between 14 and 16% [13].

On the other hand, the incidence of RC in the population at high risk of urolithiasis (2.24%) of Sector Zaragoza II in 2017 (Group A) was five times higher than in the general population of the same Sector the same year (0.46%). The following year, the incidence of RC in the general population of this Sector virtually remained the same (0.45%), whereas it halved (1.12%) in the high-risk population (Group B). This could be explained by the higher control and implementation of preventive measures and healthy habits in patients who were informed of their risk of stone formation. Apparently, these measures were effective in reducing crystal precipitation and aggregation, which are associated with the formation of kidney stones.

In agreement with our results, some studies demonstrate that crystalluria is a previous step in the formation of kidney stones and an indicator of this process. This means that a high proportion of patients with crystalluria will be stone formers [15]. Based on these findings, some authors suggest that the presence of one or two risk factors is associated with a higher risk for urolithiasis. Risk factors cause urine saturation, crystal formation and aggregation and the formation of stones, which cause clinical symptoms [4]. Patients with recurrent lithiasis exhibit larger crystals and more aggregates than non-formers. Some studies indicate that crystalluria in first-morning urine is a prognostic factor of recurrent urolithiasis [16].

The relationship between urolithiasis and some diseases, environmental and socio-demographic factors and habits has been extensively studied, especially in relation to sedentarism, inappropriate dietary habits, overweight, stress and factors such as climate and water intake [11], [12], [13], [17], [18]. Stone formers should be recommended to increase their liquid intake to reduce the saturation of some compounds in urine and prevent the formation of calculi [10]. Additionally, patients should receive dietary recommendations such as reducing the intake of proteins and sodium and maintaining an adequate intake of calcium [11], [16], [19], [20], [21], [22].

A limitation of this study is that other factors (clinical or not) may have been involved the development of RC in our population. Additionally, the analysis of urinary sediment was only performed in samples that showed alterations in the qualitative analysis of urine. Therefore, patients with crystalluria who showed normal screening results could have been missed. Only the patients with COD, COM, and UA crystals who met the criteria described above were included in the study. However, more comprehensive RC prevention strategies should also be applied to patients with other types of urine crystals such as struvite, cystine and brushite. Finally, the level of patient adherence to the recommended hygienic-dietary measures was not assessed in the study. Therefore, some patients could have not followed recommendations, which could have led to an underestimation of results. Consequently, the results of this study are preliminary and further studies are needed to obtain more detailed information on the effectiveness of hygienic-dietary recommendations in reducing the number of RC events. In future studies, patient adherence to dietary recommendations should be controlled by close individualized monitoring.

The incorporation of alerts on the risk of urolithiasis along with the inclusion of hygienic-dietary recommendations in laboratory reports may be effective in reducing the incidence of RC.
